# MANV(Massenanfall von Verletzten)-Klinikalarmierung: „Die ersten 10 Minuten“

**DOI:** 10.1007/s00113-025-01554-1

**Published:** 2025-03-27

**Authors:** Nina Thies, P. Fischer, T. Kohlmann, A. Bayer, V. Pedersen, W. Böcker, S. Prückner, M. Klein

**Affiliations:** 1 Institut für Notfallmedizin und Medizinmanagement (INM), Klinikum der Universität München, LMU München, Campus Innenstadt, Schillerstr. 53, 80336 München, Deutschland; 2 Zentrale Notaufnahme, Klinikum der Universität München, LMU München, München, Deutschland; 3 Neurologische Klinik und Poliklinik, Klinikum der Universität München, LMU München, München, Deutschland; 4 Klinik und Poliklinik für Orthopädie und Unfallchirurgie, Muskuloskelettales Universitätszentrum München (MUM), Klinikum der Universität München, LMU München, München, Deutschland; 5 Arztpraxis Wolfratshausen-Waldram, Wolfratshausen-Waldram, Deutschland

**Keywords:** Checkliste, Einsatzablauf, Alarmierungssystem, Krankenhaus Alarm- und Einsatzplan, Stufenplan, Checklist, Operation sequence, Alarm system, Disaster planning, Graduated scheme

## Abstract

**Hintergrund:**

Bei einem Massenanfall von Verletzten (MANV) müssen von der initialen Alarmierung bis zum Eintreffen des ersten Patienten in den Kliniken in kürzester Zeit Entscheidungen zu räumlicher, personeller und organisatorischer Umstrukturierung der Notaufnahme getroffen werden. Aufgrund der Seltenheit eines MANV stellt diese komplexe Aufgabe eine besondere Herausforderung dar.

**Ziel:**

Ziel der Arbeit war, die unmittelbare Vorbereitungsphase nach Auslösung eines MANV-Alarms durch konkret ausgearbeitete Handlungsanweisungen zu optimieren und die Zeit bis zur automatisierten, externen Alarmierung zu verkürzen.

**Material und Methoden:**

In einem strukturierten Dialog wurden unter Einbeziehung der Fachbereiche Notfallmedizin, Anästhesie und Chirurgie die kritischen Punkte in der Initialorganisation nach MANV-Aktivierung identifiziert und ein Flowchart für die ersten relevanten Entscheidungsprozesse erarbeitet. Die benötigte Zeit für die zu treffenden Entscheidungen wurde vor und nach Implementierung einer Handlungsanweisung „Die ersten 10 Minuten“ in praxisnahen Simulationen verglichen.

**Ergebnisse:**

Erarbeitete Checklisten „Die ersten 10 Minuten“ beinhalten eine übersichtlich strukturierte Anleitung der Aufgaben, die in den ersten Minuten von den Entscheidungsträgern vor Ort erledigt werden müssen. Durch Einführung des Konzepts konnte die Dauer des initialen Entscheidungsprozesses im Mittel um 14,3 min verkürzt werden.

**Schlussfolgerungen:**

Eine auf den Standort optimierte Entwicklung von Handlungsanweisungen mittels Checkliste führt zu einer signifikanten Beschleunigung der initialen Entscheidungsprozesse bei Aktivierung eines MANV.

**Graphic abstract:**

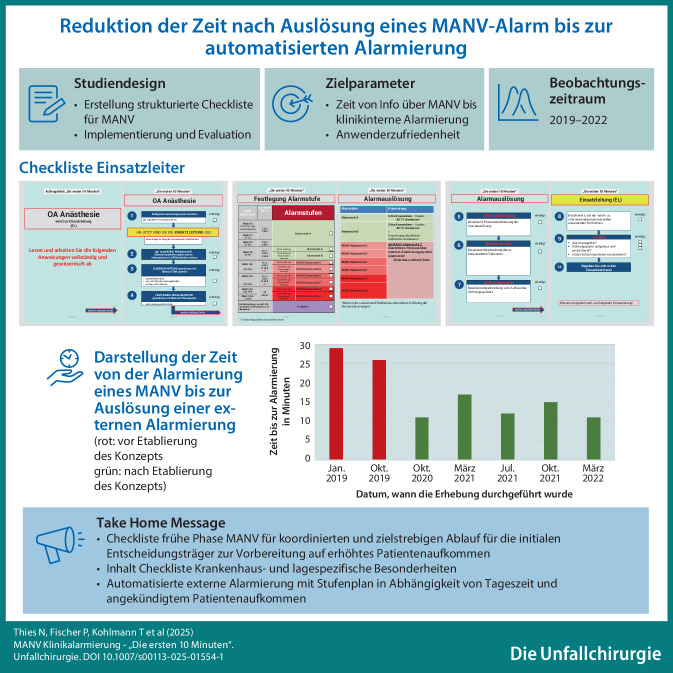

**Zusatzmaterial online:**

Die Online-Version dieses Artikels (10.1007/s00113-025-01554-1) enthält zusätzlich die Handlungsanweisungen für Oberarzt Notaufnahme und Triagekraft Notaufnahme.

## Abstract (en)

**Background:**

In the event of a mass casualty incident (MASCAL) decisions on the spatial, personnel and organizational restructuring of the emergency department have to be made in a very short time from the initial alert to the arrival of the first patient at the hospitals. Due to the rarity of a MASCAL this complex task is a special challenge.

**Aim:**

The aim of the study was to optimize the immediate preparation phase after a MASCAL alarm by means of concise instructions for action and to shorten the time to the automated external alerting.

**Material and methods:**

In a structured dialogue involving the departments of emergency medicine, anesthesia and surgery, the critical issues in the initial organization after a MASCAL activation were identified and a flow chart for the first relevant decision-making processes was developed. The time required for the decisions to be made was compared before and after the implementation of the instructions for action “The first 10 minutes” in practical simulations.

**Results:**

The compiled checklists “The first 10 minutes” contain a clearly structured guide to master the necessary tasks by decision-makers on site in the initial phase. By introducing the concept the duration of the initial decision-making process was reduced by an average of 14.3 min.

**Conclusion:**

The development of instructions for action optimized for the location with a structured checklist leads to a significant acceleration of the initial decision-making processes when a MASCAL is activated.

Bei einem Massenanfall von Verletzten (MANV) müssen Kliniken mit einer hohen Anzahl von Verletzten rechnen und sich auf eine gleichzeitige Versorgung einer größeren Anzahl von Patienten einstellen [5]. Die Eintreffzeit bis zum ersten Patienten kann nur wenige Minuten betragen. Diese knappe Zeit muss genutzt werden, um die Notaufnahme und alle anderen nachgeschalteten Strukturen sowie den Operationsbereich und die Intensivstationen für die Versorgung einer größeren Anzahl von Verletzten optimal vorzubereiten.

## Hintergrund

Bei Ereignissen mit einer hohen Anzahl von Verletzten werden die Patienten vom Rettungsdienst am Schadensort erstversorgt und vielerorts nach einem vordefinierten Verteilungsmuster in die umliegenden Kliniken gebracht, sodass eine möglichst gleichmäßige Belastung der möglichen Zielkrankenhäuser gewährleistet ist.

Bei einem Terror-MANV oder unmittelbarer Nähe zum Schadensort kann die Vorbereitungsphase, bis der erste Patient eintrifft, deutlich unkalkulierbarer und kürzer sein [3]. Zudem kann es (sowohl bei einem normalen MANV wie auch bei einem Terror-MANV) zunächst zu einem unkontrollierten Patientenzustrom durch Selbsteinweiser, die nicht durch den Rettungsdienst versorgt und gesichtet wurden, kommen. Für die Kliniken ist dadurch die Vorbereitungsphase, bis der erste Patient eintrifft, deutlich verkürzt [1, 3].

Generell gilt es, die Abläufe des Routinebetriebs sofort auf die Besonderheiten bei einem MANV anzupassen [6]. Dabei sind eine Führungsstruktur zu etablieren, die betreffenden Abteilungen zu informieren, die zentrale Notaufnahme (ZNA) für die Versorgung einer großen Anzahl von Verletzten vorzubereiten sowie – je nach Größe des Ereignisses – auch dienstfreies Personal zu alarmieren.

Die Alarmierung von dienstfreiem Personal erfolgt in der Regel über ein automatisiertes, externes Alarmierungssystem. In Abhängigkeit von der angekündigten Patientenzahl sowie personellen Ressourcen des jeweiligen Klinikums sollte die Notwendigkeit zur externen Alarmierung mittels eines Stufenplans festgelegt werden. In diesem Plan sollten in Abhängigkeit von der Größe des Klinikums verschiedene Stufen, die die Tageszeit sowie die zu erwartende Anzahl von Patienten berücksichtigen, für die Alarmierung hinterlegt sein. Eine Möglichkeit der stufenweisen Alarmierung verhindert, dass zu viel zusätzliches Personal alarmiert wird.

Gerade bei großen maximalversorgenden Kliniken sind die Handlungsabläufe und Entscheidungsprozesse in der Initialphase zunehmend komplex. Da der Personenkreis der für die Alarmierung im MANV-Fall verantwortlichen Mitarbeiter gerade in großen Kliniken in der Dienstzeit sehr variabel und mit einer hohen Fluktuation behaftet ist, ist eine regelmäßige Schulung schwierig umzusetzen. Aufgrund der Seltenheit eines MANV kommt es deshalb schnell zu Unsicherheiten in der Initiierungsphase eines MANV.

Ziel dieser Arbeit war es, die Entscheidungsträger in der Vorbereitungsphase durch eine selbsterklärende Anleitung so durch die initialen Prozessschritte zu lenken und zu instruieren, dass die externe Alarmierung und die nötigen Vorbereitungen trotz Zeitdruck und fehlender Routine vollständig und schnell durchgeführt werden konnten.

## Ausgangssituation

Das LMU Klinikum als Hochschulklinikum der Versorgungsstufe 3 mit einer Notaufnahme der umfassenden Notfallversorgung hält für seine beiden Standorte Innenstadt und Großhadern Krankenhausalarm- und Einsatzpläne (KAEP) nach Art. 8 Abs. 1 des Bayerischen Katastrophenschutzgesetzes (BayKSG) mit identischer Struktur vor; diese sind an die besonderen strukturellen, organisatorischen und personellen Gegebenheiten der beiden Standorte angepasst.

Am LMU-Klinikum setzt sich die initiale taktisch-operative Führungsstruktur (MedEL) aus 3 Schlüsselpositionen der Dienstmannschaft, die sowohl während als auch außerhalb der Regelarbeitszeit besetzt sind, zusammen. Dies sind (i) der anästhesiologische Dienstoberarzt, (ii) der chirurgische Oberarzt der ZNA und (iii) die Triage-Pflegekraft der ZNA. Diese 3 Positionen werden unmittelbar nach der Alarmierung zusammengerufen und finden sich umgehend in der ZNA ein. Sie entscheiden je nach angekündigter Patientenzahl bzw. MANV-Stufe und Tageszeit, ob eine Umstrukturierung vom Routinebetrieb auf den MANV-Betrieb vorgenommen wird, in welchem Umfang dies ggf. erfolgen soll – und koordinieren entsprechend alle notwendigen Aufgaben. Für diese Entscheidungen sind Algorithmen hinterlegt.

Um die Initialphase der Umstrukturierung und Anpassung der Abläufe auf die MANV-Versorgung zu trainieren, werden am LMU Klinikum in regelmäßigen Abständen Übungen durchgeführt. Diese Übungen werden während des laufenden Betriebs vom Institut für Notfallmedizin und Medizinmanagement (INM) organisiert und in den zentralen Notaufnahmen durchgeführt. Das Resultat erster Übungen hatte gezeigt, dass es vor Einführung des hier vorgestellten Konzepts im Mittel bis zu 27,5 min dauerte, bis das dienstfreie Personal über ein automatisiertes, externes Alarmierungssystem verständigt wird. Dieser relativ große Zeitverzug war der Auslöser für die Entwicklung des Konzepts „Die ersten 10 Minuten“.

## Methodenteil

Die Entwicklung des Konzepts einer Strukturierung von der Vorbereitungsphase bis zum Eintreffen des ersten Patienten bei einem MANV wurde in 3 definierten Schritten auf Grundlage eines Expertenkonsenses erstellt.

Im ersten Schritt wurde durch Mitarbeiter des INM ein erster Entwurf der Checkliste „Die ersten 10 Minuten“ für die 3 initialen Entscheidungsträger (i) anästhesiologischer Dienstoberarzt (später Einsatzleiter), (ii) Oberarzt ZNA (später Einsatzabschnittsleiter Notaufnahme) und (iii) Pflege Notaufnahme entwickelt. Dabei wurde zunächst festgelegt, welche Aufgaben in den ersten 10 min durchgeführt bzw. welche Entscheidungen getroffen werden müssen, um die Klinik vom Routinebetrieb adäquat auf die Abarbeitung eines MANV-Szenarios vorzubereiten.

Anfang Mai 2019 wurde der Leitung der ZNA und der Anästhesie der erste Entwurf vorgestellt und auf inhaltliche Konsistenz und formale Kriterien überprüft. In einem weiteren Termin Ende Mai 2019 wurde das Konzept durch einen erweiterten Kreis von Fachvertretern aus Anästhesie und Chirurgie beurteilt und abgestimmt. Im Juli 2019 wurde das Konzept auf einem Treffen mit allen relevanten Fachdisziplinen (ZNA, Pflege, Unfallchirurgie, Viszeralchirurgie, Anästhesie) konsentiert.

In einem weiteren Schritt wurden die Checklisten im Rahmen von ersten Testläufen während des Klinikbetriebs auf Praktikabilität überprüft und kleinere Anpassungen vorgenommen. Dazu wurde den diensthabenden Personen, die bei einem MANV die Adressaten des Konzepts „Die ersten 10 Minuten“ sind, die jeweils aktuelle Version der Checkliste vorgelegt. Ziel war es, diese durchzuarbeiten und eine Rückmeldung zu erhalten, an welchen Stellen Formulierungen missverständlich sind, um diese nochmals eindeutiger zu formulieren. Insgesamt wurden von Juli bis August 2019 drei Testläufe durchgeführt.

## Ergebnisse

### Struktur des Konzepts „Die ersten 10 Minuten“

Die Checkliste „Die ersten 10 Minuten“ wurde in Form eines Flussdiagramms erstellt und grundsätzlich so konzipiert, dass sie inhaltlich auch ohne Schulung abzuarbeiten ist. Es ist jede abzuarbeitende Aufgabe in einem Kasten beschrieben. Die Kästen sind untereinander angeordnet, sodass die strukturierte Abarbeitung von oben nach unten erfolgt, zusätzlich unterstützt durch eine aufsteigende Nummerierung (Abb. 1).

**Abb. 1:**
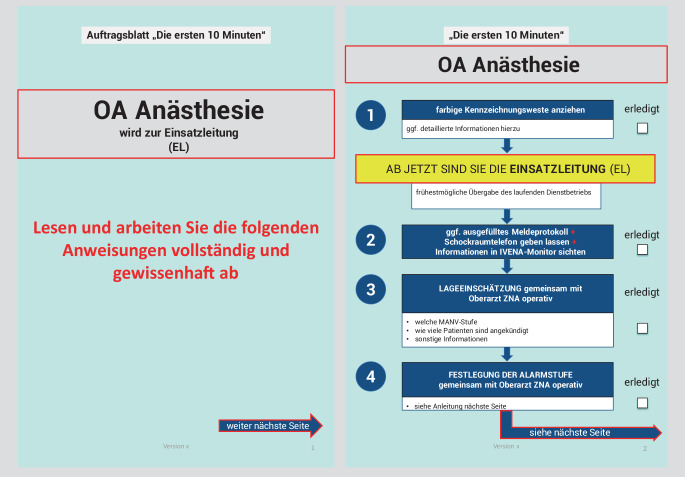
Handlungsanweisung „Die ersten 10 Minuten“ am Beispiel des Oberarztes der Anästhesie (Einsatzleiter)

**Abb. 1:**
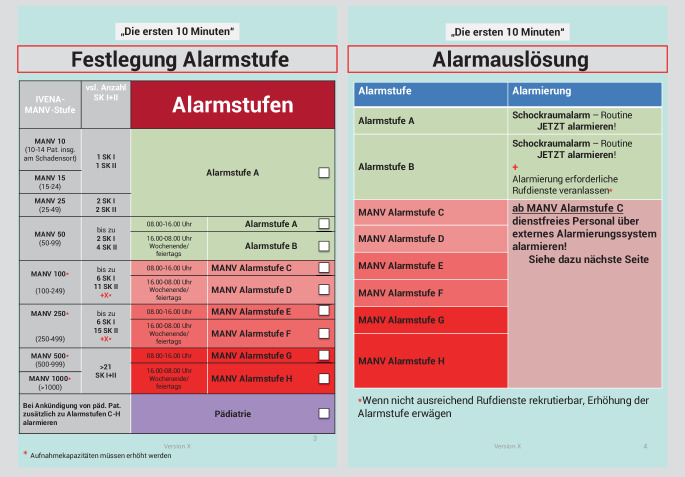
Fortführung der Handlungsanweisung „Die ersten 10 Minuten“: Festlegung der Alarmstufe und Alarmauslösung

**Abb. 1:**
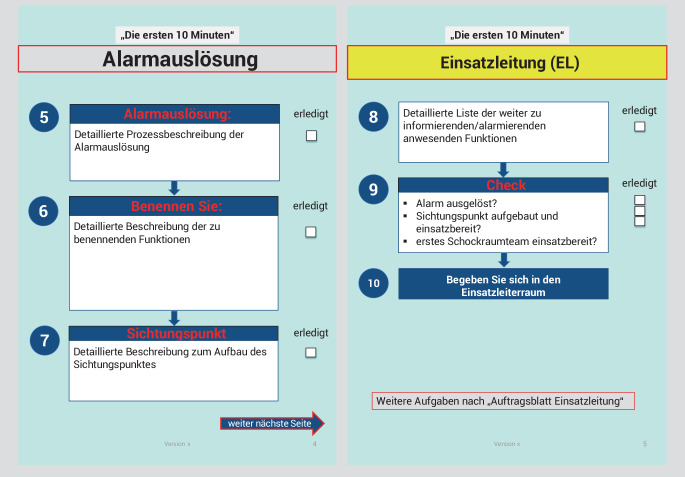
Fortführung der Handlungsanweisung „Die ersten 10 Minuten“: Alarmauslösung und Einsatzleitung

Das jeweilige Titelblatt der verschiedenen Handlungsanweisungen für die 3 Entscheidungsträger enthält den Adressaten der Handlungsempfehlung, also (i) Oberarzt Anästhesie, (ii) Oberarzt ZNA und (iii) Pflege ZNA. Die folgende Seite definiert die Funktion, die die 3 initialen Entscheidungsträger im MANV übernehmen müssen, und die nächsten Schritte, die im Workflow durchzuführen sind (Abb. 1). Die Handlungsanweisungen für die 3 Entscheidungsträger unterscheiden sich in einzelnen Punkten, um Redundanzen zu vermeiden und die Effizienz des Entscheidungsteams zu erhöhen.

Im Detail werden folgende Aufgaben in der Checkliste aufgeführt: 1. Kennzeichnung mittels farbiger Kennzeichnungsweste der neuen eigenen individuellen Funktion im MANV z. B. Oberarzt Anästhesie wird Einsatzleiter, 2. und 3. Lageeinschätzung (Welche Stufe auf der Patientenverteilmatrix liegt vor? Wie viele Patienten sind angekündigt? Gibt es weitere wichtige Informationen z. B. bezüglich des Szenarios? Liegt eine statische oder dynamische Lage vor? Sind pädiatrische Patienten zu erwarten?), 4. Festlegung der Alarmstufe klinikinterne bzw. externe Alarmierung (S. 3 und 4 in Abb. 1 des Supplements kann der Tabelle in Abhängigkeit von der Tageszeit und der angekündigten Patientenzahl entnommen werden, wer alles informiert werden muss), 5. Anleitung zur externen Alarmauslösung, 6. Benennung weiterer essenzieller Führungspersonen laut Organigramm in Abhängigkeit von der festgelegten Klinikalarmstufe, 7. Umrüstung der zentralen Notaufnahme für den MANV-Betrieb mit einer detaillierte Auflistung was gemacht werden muss z. B. Sichtungspunkt aufbauen 8. Liste von anwesender Mitarbeiter im Klinikum, die informiert werden müssen 9. Auflistung der wichtigsten Punkten der Checkliste, um sicherzustellen, dass keine essentiellen Aufgaben vergessen wurden wie z. B. die Alarmierung des ersten Schockraumteams, das Aufbauen des Sichtungspunktes oder die Alarmierung von dienstfreiem Personal (Abb. 1 sowie Zusatzmaterial online).

### Implementierung des Konzepts „Die ersten 10 Minuten“

Die anästhesiologischen Oberärzte, die bei einem realen Szenario die Aufgabe des Einsatzleiters übernehmen, wurden im neuen Konzept „Die ersten 10 Minuten“ geschult. Drei Schulungen wurden im September 2019 durchgeführt sowie eine weitere im August 2020. Insgesamt konnten dadurch *n* = 28 Oberärzte geschult werden.

Die Qualifikationen der anästhesiologischen Oberärzte setzte sich breit zusammen, sowohl bezüglich der Erfahrung als auch des Alters. Alle Teilnehmer verfügten über den Facharzt Anästhesiologie und waren als Oberärzte tätig. Vierzehn Teilnehmer konnte eine Berufserfahrung bis zu 5 Jahren als Oberarzt vorweisen. Acht Teilnehmer konnte eine Erfahrung bis zu 10 Jahren und 6 über 10 Jahre bieten.

Bei allen Teilnehmern wurde jeweils eine Erstunterweisung des neuen Konzepts durchgeführt.

Zur abschließenden Implementierung (am 07.10.2020) und zur regelmäßigen Überprüfung des Konzepts „Die ersten 10 Minuten“ wurden Übungen mit folgendem Ablauf durchgeführt: (i) simulierter Anruf der Integrierten Leitstelle, welcher die Klinik über einen MANV sowie über die Übungssituation informiert. (ii) Die initialen Entscheidungsträger wurden informiert und haben die neu erstellten Checklisten erhalten. (iii) Die Checklisten wurden bearbeitet wie bei einer realen Alarmierung. (iv) Die Übung wurde beendet, sobald jeder der 3 Funktionsträger seine Checkliste vollständig abgearbeitet hatte.

Der Ablauf der Übungen wurde identisch durchgeführt. Die Übungsszenarien wurden bezüglich Übungszeitpunkt und Anzahl der zu erwarteten Verletzten leicht variiert. Alle Szenarien wurden so ausgelegt, dass alle Inhalte des neuen Konzepts umgesetzt werden mussten und das dienstfreie Personal alarmiert werden musste.

Das Kernszenario wurde wie folgt beschrieben: „Es hat sich ein Großschadensfall mit vielen Verletzten ereignet. Es handelt sich um eine Kollision von zwei S‑Bahnen. Ihre Klinik ist für die Aufnahme von Schwerverletzen im Rahmen der Krankenhausalarmierung gemäß Patientenverteilmatrix vorgesehen. Es wird die ^**^Patientenverteilmatrix (PVM) Stufe XXX^**^ ausgelöst. Bitte treffen Sie die nötigen Vorbereitungen.“

Die Zeit von der fiktiven Alarmierung bis zur Auslösung der externen Alarmierung von Personal wurde dokumentiert (Tab. 1). Im Mittel dauerte es bis zur Alarmierung ohne Verwendung des Konzepts 27,5 min, was mit Nutzung des neuen Konzepts auf 13,2 min reduziert werden konnte.

**Tab. 1:** Dokumentation der Zeit bei den durchgeführten Übungen (von der Information der ZNA über die MANV Lage bis zur Auslösung der externen Alarmierung von Personal)

Übung am	Zeit von Information der ZNA über die MANV Lage bis zur Auslösung der automatisierten, externen Alarmierung von Personal
*31.01.2019*	*29 min (ohne das Konzept „Die ersten 10 Minuten“)*
*07.10.2019*	*26 min (ohne das Konzept „Die ersten 10 Minuten“)*
07.10.2020	11 min (mit dem Konzept „Die ersten 10 Minuten“)
30.03.2021	17 min (mit dem Konzept „Die ersten 10 Minuten“)
20.07.2021	12 min (mit dem Konzept „Die ersten 10 Minuten“)
12.10.2021	15 min (mit dem Konzept „Die ersten 10 Minuten“)
22.03.2022	11 min (mit dem Konzept „Die ersten 10 Minuten“)

Am 30.03.2021 wurden zunächst veraltete Dokumente ohne Checklistenformat zur Auslösung der externen Alarmierung genutzt, was die deutlich verlängerte Zeit vom simulierten Anruf der Leitstelle bis zur erfolgreichen externen Alarmierung erklärt

## Diskussion

Um die strukturierte und schnelle Abarbeitung eines MANV in einer Klinik zu ermöglichen, reicht es nicht aus, einfach nur einen KAEP vorzuhalten. In der vorliegenden Arbeit wurde die Festlegung eines Alarmierungsschemas für eine Klinik der Maximalversorgung im Falle eines MANV erarbeitet, in einer strukturierten Handlungsanweisung festgehalten, etabliert und mittels Praxistest evaluiert.

Die Ergebnisse unserer Übung zur Vorbereitungsphase zeigen, dass die Zeit von der Alarmierung durch die Leitstelle bis zur Auslösung des klinikinternen Alarms durch das Konzept „Die ersten 10 Minuten“ um 50 % reduziert werden konnte.

Als Ursachen für die initial lange Zeit bis zur Alarmierung konnten in mehreren Qualitätszirkeln unterschiedliche Punkte identifiziert werden: Zum einen bestand Unklarheit darüber, ab wann eine Alarmierung von externem Personal durchgeführt werden sollte. Aufgrund der Größe des Klinikums und der Menge der in einem MANV-Fall bei externer Alarmierung automatisch informierten Mitarbeiter bestand eine große Verunsicherung, den Schritt zu einer externen Alarmierung tatsächlich zu gehen. Zudem stellte sich heraus, dass aufgrund der Seltenheit eines MANV-Ereignisses alle Mitarbeiter zwar bezüglich der Existenz eines MANV-Alarmierungsplans informiert waren, über die Details in der Initialphase bezüglich Alarmierung jedoch nicht ausreichend vertraut waren und die Entscheidungsprozesse ad hoc nicht oder nur schwer umsetzen konnten.

Der Zeitdruck, in möglichst kurzer Zeit die klinischen Strukturen vom Routinebetrieb auf die Bewältigung eines MANV umzuwandeln, bedeutet für die initialen Entscheidungsträger immer enormen Stress. In zahlreichen Arbeiten zu Human Factors wird beschrieben, dass der Effekt von Stress zu eingeschränkter Aufmerksamkeit, eingeschränktem Urteils- und Entscheidungsvermögen sowie eingeschränkter Gedächtnisleistung führt, wohingegen ein moderater Stresslevel die Leistungsfähigkeit erhöhen kann [4]. Um das Stresslevel der initialen Entscheidungsträger bei einem MANV möglichst im moderaten Bereich zu halten, ist es entscheidend, die Vorbereitungsphase zu unterstützen. Die Reduktion der Zeit bis zur klinikinternen Alarmierung durch die neu erarbeiteten Checklisten führt zu einer deutlichen Stressreduktion der Entscheidungsträger und gibt die Möglichkeit, mit diesem Konzept strukturiert die ersten Entscheidungen gemeinsam im Team mithilfe von Checklisten treffen zu können. Zusätzlich wurde beobachtet, dass Fehler in der gestuften Alarmierung in Abhängigkeit vom gemeldeten Szenario nach Einführung der Checklisten „Die ersten 10 Minuten“ weniger häufig auftraten, wobei dieser Aspekt nicht systematisch untersucht wurde.

Thomassen et al. konnten in einem systematischen Review feststellen, dass die Patientensicherheit in der Medizin mithilfe von Checklisten, u. a. durch Berücksichtigung der Human-Factor-Aspekte, verbessert werden kann. Zu den Human-Factor-Aspekten, die für Patientensicherheit relevant sind, gehört eine Veränderung der Kommunikation, der Führung, der Koordination, das Bewusstsein für die Situation sowie das Teilen eines mentalen Models [7]; all diese Aspekte sind auch auf die Bewältigung eines MANV übertragbar.

Aus der Umfrage von Fischer et al. „Wie gut sind Ärzte auf einen Massenanfall von Verletzten vorbereitet?“ geht hervor, dass nur 42,7 % der Teilnehmer eine Übung im Umgang mit einem MANV absolviert hatten [2].

Flächendeckende Übungen stellen in Deutschland noch eine Seltenheit dar. In der Arbeit von Schweigkofler et al. über die Schulung bei einem MANV wird zwar eine flächendeckende Übung seitens der Bundesländer gefordert [6], die Umsetzung in der Praxis ist jedoch nicht absehbar. Infolgedessen erscheint es essenziell, die Prozesse so zu gestalten, dass möglichst wenig Schulung notwendig ist, um eine fehlerfreie Umsetzung zu gewährleisten.

Zusammenfassend kann festgehalten werden, dass eine optimale und schnelle klinikinterne Vorbereitungsphase bei einem MANV einer besonderen Struktur bedarf. Diese Struktur kann schnell und einfach durch Checklisten für initialen Entscheidungsträger aufgebaut werden. Die Checklisten enthalten klar formulierte und logisch aufeinander aufbauende Handlungsanweisungen für eine vollständige Durchführung aller erforderlichen Schritte. Dadurch kann der Stress der initialen Entscheidungsträger reduziert werden.

## Fazit

- Nach der Information eines Krankenhauses über einen MANV muss die Vorbereitungsphase für die initialen Entscheidungsträger klar strukturiert sein.
- Für den koordinierten Ablauf der ersten Phase kann eine Checkliste für die initialen Krankenhaus- und lagespezifischen Besonderheiten hilfreich sein.
- Es empfiehlt sich, die Überprüfung der Praktikabilität im Rahmen von Übungen durchzuführen.
- Eine automatisierte, externe Alarmierung sollte mittels eines Stufenplans in Abhängigkeit von Tageszeit und angekündigter Patientenzahl etabliert werden.

## Supplementary Information

Handlungsanweisungen Oberarzt Notaufnahme und Triagekraft Notaufnahme

## Data Availability

Die erhobenen Datensätze sind im Artikel oder im Zusatzmaterial online zu finden.
