# Supplementary material for: MANSCAL(mass casualty incident) hospital alert: “The first 10 minutes”
Source: Unfallchirurgie (Heidelb). 2025 Mar 27;128(6):441–8. [Article in German] doi: 10.1007/s00113-025-01554-1 (PMC12116604; doi:10.1007/s00113-025-01554-1)
Supplement: Supplementary file 1 — Handlungsanweisungen Oberarzt Notaufnahme und Triagekraft Notaufnahme [file 113_2025_1554_MOESM1_ESM.pdf]

Supplementary Information

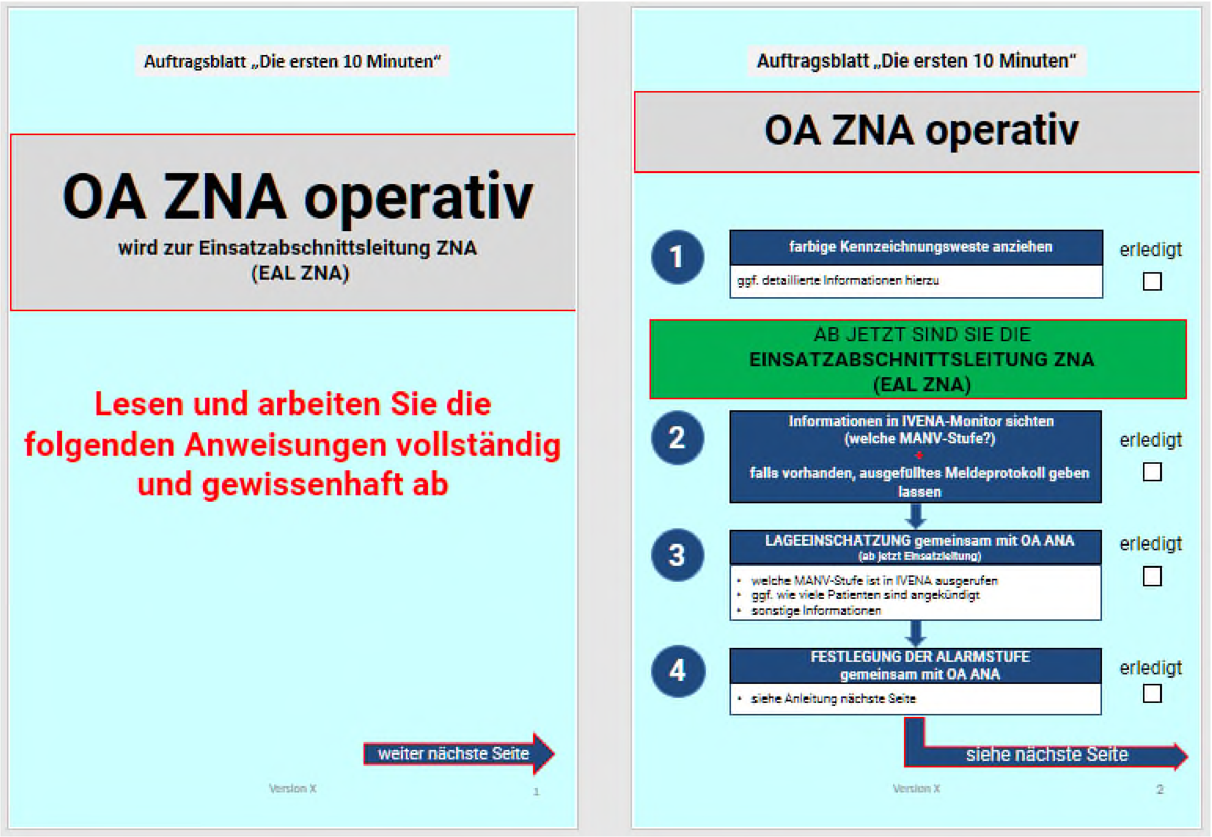

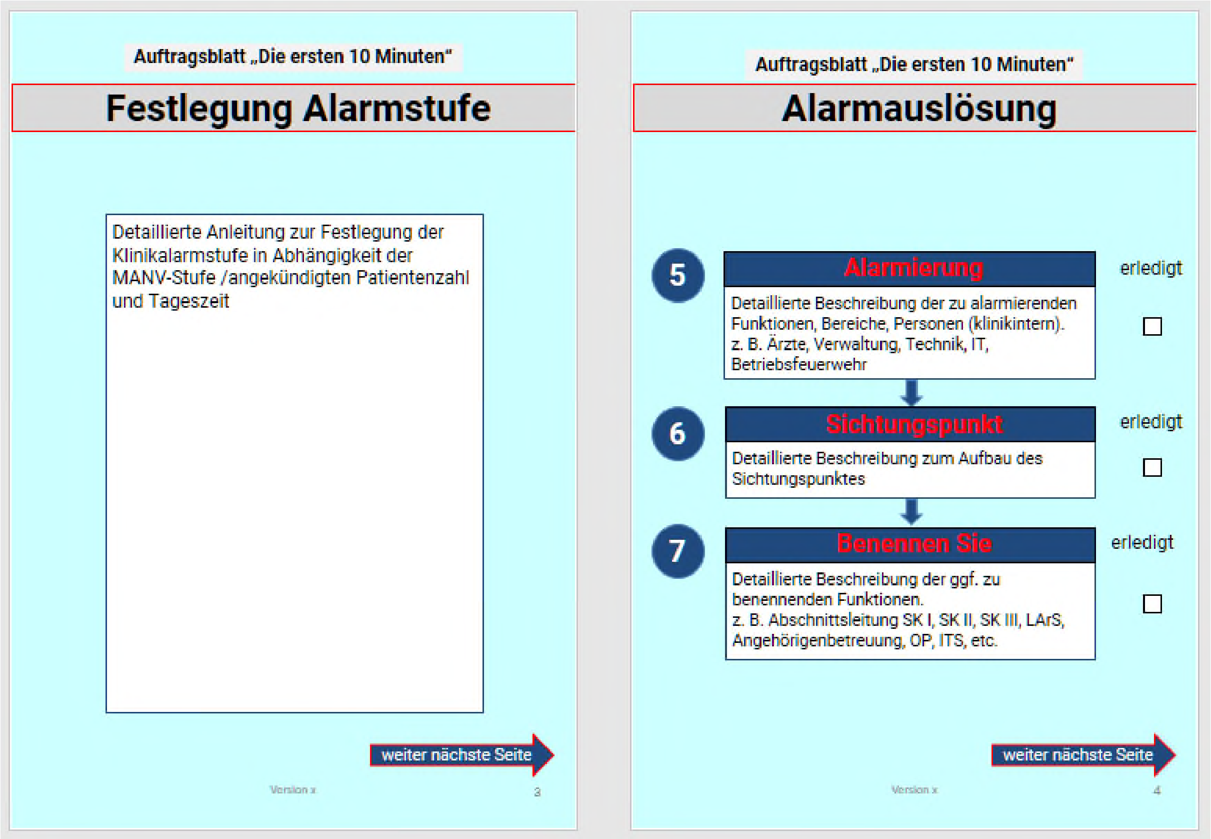

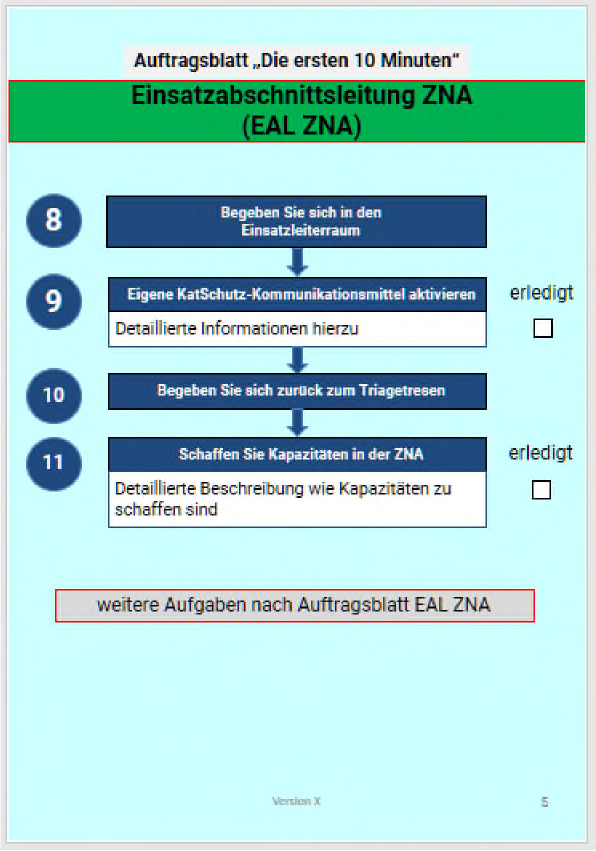

Abb.2. Handlungsanweisung „Die ersten 10 Minuten" am Beispiel des Oberarztes Notaufnahme (Einsatzabschnittsleitung ZNA)

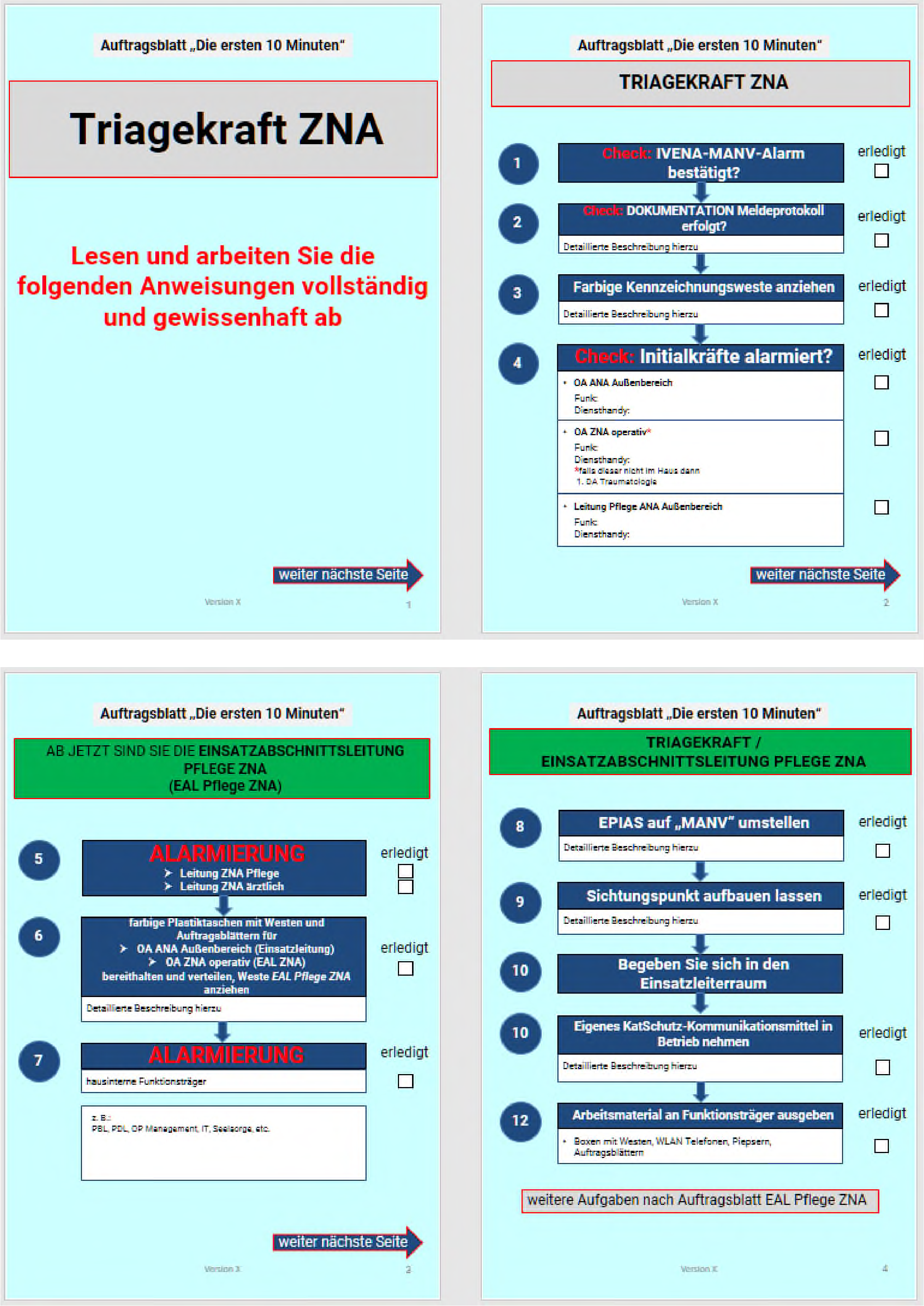

Abb.3. Handlungsanweisung „Die ersten 10 Minuten“ am Beispiel der Triagekraft Notaufnahme (Einsatzleiterabschnittsleitung Pflege ZNA)
